# 9,10-Dioxa-1,2-diaza-anthracene derivatives from tetrafluoropyridazine

**DOI:** 10.3762/bjoc.6.45

**Published:** 2010-05-06

**Authors:** Graham Pattison, Graham Sandford, Dmitrii S Yufit, Judith A K Howard, John A Christopher, David D Miller

**Affiliations:** 1 Department of Chemistry, University of Durham, South Road, Durham, DH1 3LE, United Kingdom; 2 Chemical Crystallography Group, Department of Chemistry, University of Durham, South Road, Durham, DH1 3LE, U.K.; 3 GlaxoSmithKline R&D, Medicines Research Centre, Gunnels Wood Road, Stevenage, Hertfordshire, SG1 2NY, United Kingdom

**Keywords:** benzodioxinopyridazine, 9,10-dioxa-1,2-diaza-anthracene, heterocyclic synthesis, nucleophilic aromatic substitution, perfluoroheteroaromatic, tetrafluoropyrazine

## Abstract

Reaction of tetrafluoropyridazine with catechol gives a tricyclic 9,10-dioxa-1,2-diaza-anthracene system by a sequential nucleophilic aromatic substitution ring annelation process, further extending the use of perfluoroheteroaromatic derivatives for the synthesis of unusual polyfunctional heterocyclic architectures. The tricyclic scaffold reacts with amines and sodium ethoxide providing a short series of functional 9,10-dioxa-1,2-diaza-anthracene systems.

## Introduction

Drug discovery programmes are continually searching for viable synthetic routes to highly novel classes of heterocyclic compounds with the aim of exploring chemical ‘drug-like’ space [1] and uncovering valuable biological activity for hit-to-lead generation of new chemical entities by parallel synthesis techniques. The wide variety of relatively simple heterocyclic structural types that have not been synthesised [2], the relatively low level of structural diversity in all known organic structures [3] and, indeed, the perceived lack of structural diversity in pharmaceutical companies’ compound collections have often been suggested to be among the bottlenecks in drug discovery programmes [4]. Methodology for the ready synthesis of new organic frameworks is still required and, in this context, heterocyclic scaffolds based on novel molecular architecture that bear multiple functionality and can be rapidly processed into many analogues by parallel synthesis are particularly valuable [5–6].

**Scheme 1:**
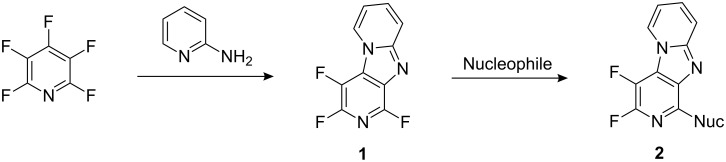
Synthesis of novel tricyclic heterocycles from pentafluoropyridine.

In a continuing research programme, we have demonstrated that perfluorinated heteroaromatic derivatives are very useful starting scaffolds for the synthesis of a variety of heteroaromatic [7], [5,6] and [6,6]-bicyclic [8–11], and polycyclic heterocyclic systems [12]. Perfluoroheteroaromatic derivatives are either commercially available or can be accessed by halogen-exchange processes by reaction of the corresponding perchloroheteroaromatic system and potassium fluoride [13]. No special techniques for handling perfluoroheteroaromatic compounds are required, apart from the usual laboratory precautions, because these systems are generally volatile, colourless liquids. We established that highly novel tricyclic scaffolds, such as the relatively uncommon dipyrido[1,2-*a*:3′,4′-*d*]imidazole system **1**, could be synthesised from pentafluoropyridine in a single step [12], exemplifying our general strategy for the synthesis of highly novel classes of polyfunctional heterocyclic compounds. Several dipyrido[1,2-*a*:3′,4′-*d*]imidazole analogues **2** were prepared by the displacement of the remaining ring fluorine atoms by nucleophilic aromatic substitution processes (Scheme 1).

**Scheme 2:**
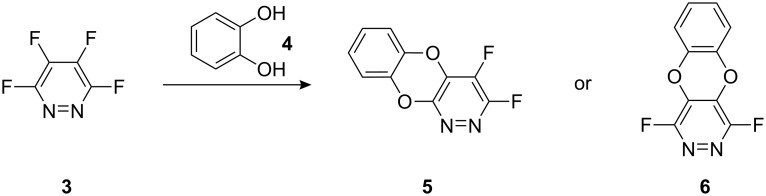
Synthetic route to dioxa-diaza-anthracene derivatives.

We were interested in further expanding the use of highly fluorinated heterocycles for the preparation of novel heterocyclic structures and focussed upon the synthesis of ring fused systems that could be derived from the reaction of tetrafluoropyridazine (**3**) with catechol (**4**). In principle, two possible systems **5** and **6** may be formed depending upon the regioselectivity of the nucleophilic aromatic substitution processes (Scheme 2).

Both **5** and **6** have ring fluorine atoms present that may, in principle, be displaced by nucleophiles which could lead to the synthesis of many analogues of these systems. The dioxa-1,2-diaza-anthracene (or 3,4-difluorobenzo[5,6][1,4]dioxino[2,3-*c*]pyridazine also referred to as benzodioxinopyridazine) systems are very rare heterocyclic structures and only a handful of analogues based upon this molecular skeleton have been synthesised, mainly by the reaction of chlorinated pyridazines with catechol [14–16].

In this paper, we describe the synthesis of dioxa-1,2-diaza-anthracene derivatives by the sequential reaction of commercially available tetrafluoropyridazine with catechol, and a short series of nucleophiles.

## Results and Discussion

**Scheme 3:**
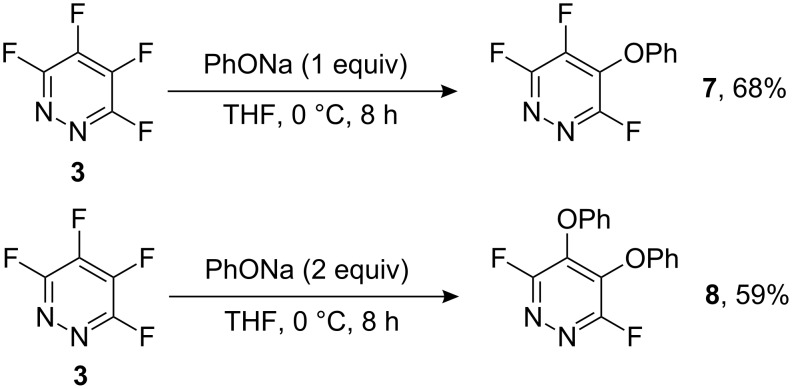
Reactions of tetrafluoropyridazine **3** with sodium phenoxide.

Initially, we carried out reactions of tetrafluoropyridazine (**3**) with one and two equivalents of sodium phenoxide as a model substrate for catechol (Scheme 3).

Reaction of one equivalent of sodium phenoxide with (**3**) gave product **7** arising from substitution of fluorine located at the site *para* to activating ring nitrogen, consistent with earlier studies involving reactions between tetrafluoropyridazine and various nucleophiles [13]. Similarly, reaction of two equivalents of sodium phenoxide gave the 4,5-diphenoxy derivative **8** by displacement of both fluorine atoms that are attached to the sites *para* to ring nitrogen atoms.

**Scheme 4:**
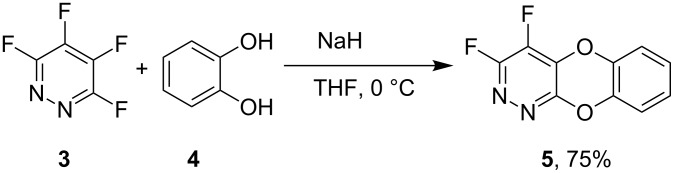
Synthesis of dioxa-1,2-diaza-anthracene scaffold **5**.

In contrast, however, reaction of catechol (**4**) with tetrafluoropyridazine (**3**) under similar reaction conditions gave the tricyclic system **5** arising from displacement of the 3- and 4-fluorine atoms as the sole product according to a ^19^F NMR analysis of the crude reaction mixture (Scheme 4). The ^19^F NMR displays two resonances at −96.4 and −151.9 ppm in accord with structure **5**, whereas if the symmetrical 4,5-disubstituted product **6** had been formed only one resonance in the ^19^F NMR spectrum at ca. −88 ppm (cf. **8**) would have been observed.

**Scheme 5:**
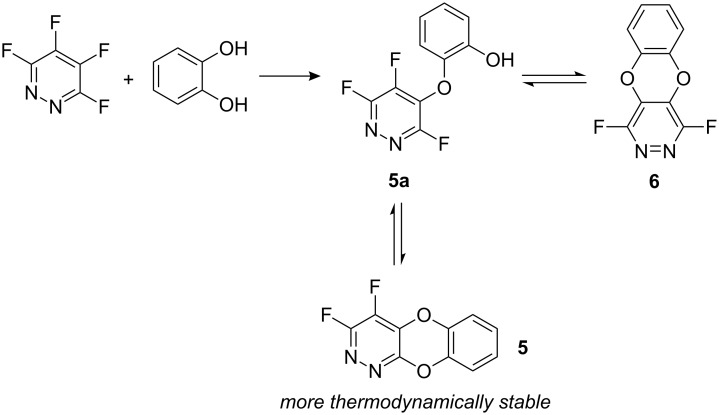
Mechanism of formation of **5**.

It seems reasonable to assume that initial substitution occurs at the 4-position of **3**, analogous to the reaction between **3** and phenoxide, to give intermediate **5a**. At this point, we would expect cyclisation to occur at position 5 to give product **6**, again by analogy to the outcome of reaction between **3** and excess phenoxide. However, since nucleophilic aromatic substitution reactions are frequently reversible [13], conversion of **6** must occur via intermediate **5a** and lead to the most thermodynamically stable product **5** (Scheme 5).

**Scheme 6:**
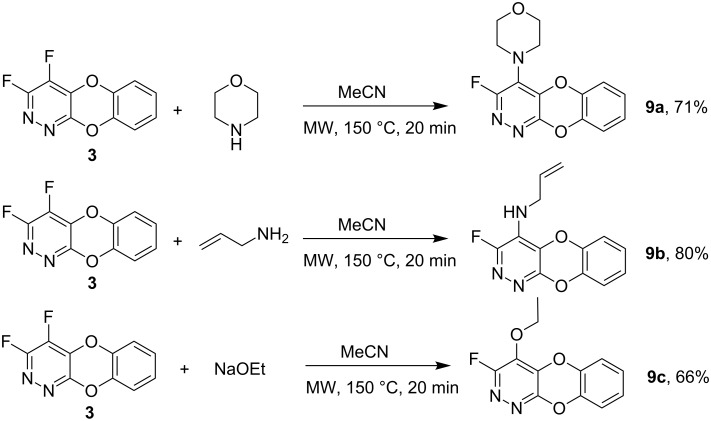
Reactions of dioxa-1,2-diaza-anthracene scaffold **5** with nucleophiles.

The utility of the dioxa-1,2-diaza-anthracene system **5** as a scaffold for array synthesis was assessed in representative reactions with a short series of nucleophiles (Scheme 6).

**Figure 1:**
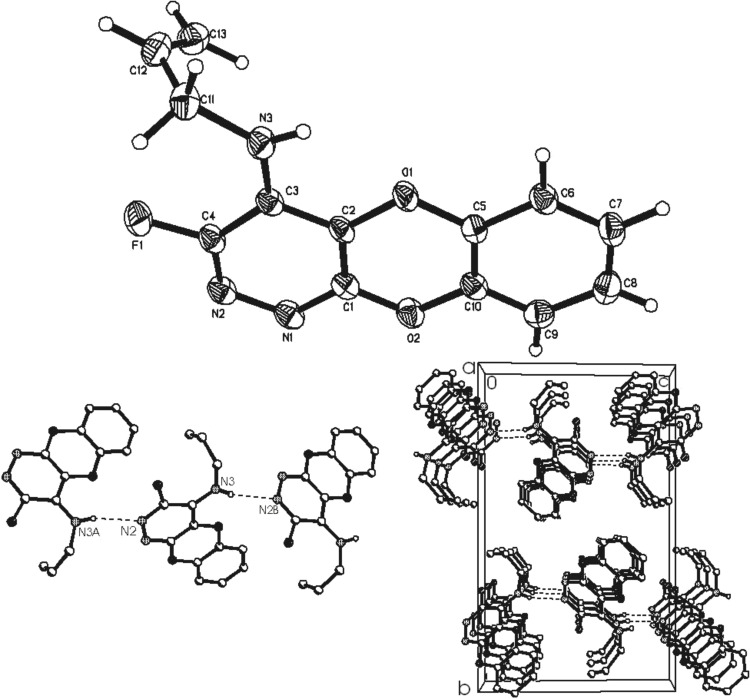
Molecular structure of 4-allylamino-3-fluoro-9,10-dioxa-1,2-diaza-anthracene (**9b**).

Nucleophilic substitution of fluorine at the 4-position occurs regiospecifically to afford products **9a–c** according to ^19^F NMR analysis of the corresponding reaction mixtures. The ^19^F NMR resonances located at ca. −90 ppm are characteristic of fluorine atoms located at sites *ortho* to a ring nitrogen atom. X-ray crystallography of the allylamino derivative **9b** (Figure 1), and a comparison of NMR spectral data, confirms the structures of these analogues.

The geometrical parameters of the molecule **9b** are close to expected values. The molecules of **9b** in the crystal are linked together by N–H···N hydrogen bonds in chains, parallel to the [101] direction and π···π stacking interactions (shortest interatomic distance C5···C3 is 3.336 Å), and short C–H···O contacts (C···O 3.387 Å) bind adjacent chains in the [100] and [010] directions, respectively.

Again, the regiospecificity of these reaction processes occurs because of the activating effect of ring nitrogen directly opposite the site of nucleophilic substitution.

## Conclusions

A small range of dioxa-1,2-diaza-anthracene analogues **5** and **9** have been synthesised from tetrafluoropyridazine in two efficient steps, further expanding the application of highly fluorinated heterocycles for the synthesis of rare heterocyclic architectures.

## Experimental

Synthetic procedures for the preparation of all the new compounds described in this paper are given below.

### Reactions of tetrafluoropyridazine (**3**) with sodium phenoxide

#### 3,4,6-Trifluoro-5-phenoxypyridazine (**7**)

Phenol (0.17 g, 1.81 mmol) was dissolved in THF (20 mL) and added to sodium hydride (0.07 g, 1.8 mmol, 60% dispersion in mineral oil) which was cooled to 0 °C and stirred. Tetrafluoropyridazine (**3**) (0.25 g, 1.64 mmol) was added slowly and the mixture stirred at 0 °C for 8 h. The solvent was evaporated and the crude material partitioned between dichloromethane (25 mL) and water (25 mL). The organic layer was separated and the aqueous layer extracted with dichloromethane (3 × 25 mL). The combined organic extracts were then dried (MgSO_4_), filtered and evaporated in vacuo to provide a crude yellow material. Column chromatography on silica gel using hexane:ethyl acetate (4:1) as elutant gave 3,4,6-trifluoro-5-phenoxypyridazine (**7**) (0.25 g, 68%) as a colourless oil; Anal. Calcd for C_10_H_5_F_3_N_2_O: C, 53.1; H, 2.2; N, 12.4%. Found: C, 53.2; H, 2.5; N, 12.2. ^1^H NMR (200 MHz, CDCl_3_, δ_H_): 7.04–7.42 (5H, m, ArH); ^19^F NMR (188 MHz, CDCl_3_, δ_F_): −86.2 (1F, dd, ^5^*J*_FF_ = 31.2 Hz, ^4^*J*_FF_ = 23.7 Hz, F-6), −94.5 (1F, dd, ^5^*J*_FF_ = 31.2 Hz, ^3^*J*_FF_ = 23.7 Hz, F-3), −140.4 (1F, dd, ^3^*J*_FF_ = 23.7 Hz, ^4^*J*_FF_ = 23.7 Hz, F-4); MS (ES^+^) *m/z*: 227 ([MH]^+^, 100%).

#### 3,6-Difluoro-4,5-diphenoxypyridazine (**8**)

Using the procedure described above, phenol (0.32 g, 3.45 mmol), sodium hydride (0.138 g, 3.45 mmol, 60% dispersion in mineral oil), tetrafluoropyridazine (0.25 g, 1.64 mmol) and THF (20 mL) gave 3,6-difluoro-4,5-diphenoxypyridazine (**8**) (0.29 g, 59%) as a white solid; mp 123–124 °C; Anal. Calcd for C_16_H_10_F_2_N_2_O_2_: C, 64.0; H, 3.4; N, 9.3%. Found: C, 63.7; H, 3.5; N, 9.2. ^1^H NMR (700 MHz, CDCl_3_, δ_H_): 6.80 (2H, d, ^3^*J*_HH_ = 7.7, H-2′), 7.09 (1H, t, ^3^*J*_HH_ = 7.7, H-4′), 7.23 (2H, t, ^3^*J*_HH_ = 7.7, H-3′); ^13^C NMR (175 MHz, CDCl_3_, δ_C_): 116.4 (s, C-2′), 124.9 (s, C-4′), 129.7 (s, C-3′), 137.2 (dd, ^2^*J*_CF_ = 20.6, ^3^*J*_CF_ = 12.9, C-4), 155.2 (s, C-1′), 160.1 (dd, ^1^*J*_CF_ = 251.2, ^4^*J*_CF_ = 6.8, C-3); ^19^F NMR (658 MHz, CDCl_3_ δ_F_): −88.2 (s); MS (ES^+^) *m/z*: 301 ([MH]^+^, 100%).

#### Synthesis of 3,4-difluoro-9,10-dioxa-1,2-diaza-anthracene (**5**)

Catechol (0.80 g, 7.2 mmol) was dissolved in THF (20 mL) at 0 °C under an argon atmosphere with stirring and added to sodium hydride (0.35 g, 14.5 mmol, 60% dispersion in mineral oil). Tetrafluoropyridazine (1.00 g, 6.6 mmol) was added dropwise and the mixture stirred at 0 °C for 8 h. After this period, the solvent was evaporated, and the crude material redissolved in dichloromethane (25 mL) and water (25 mL). The organic layer was separated and the aqueous layer extracted with dichloromethane (3 × 25 mL). The combined organic extracts were then dried (MgSO_4_), filtered and evaporated to provide a crude yellow material. Crystallisation from acetonitrile gave 3,4-difluoro-9,10-dioxa-1,2-diaza-anthracene (**5**) (1.09 g, 75%) as white solid; mp 146–148 °C; Anal. Calcd for C_8_H_11_FN_4_O: C, 54.1; H, 1.8; N, 12.6%. Found: C, 54.0; H, 1.9; N, 12.6. IR, ν_max_/cm^−1^: 1015, 1035, 1094, 1115, 1260, 1416, 1464, 1490, 1568, 1654; ^1^H NMR (400 MHz, CDCl_3_, δ_H_): 7.13–7.06 (4 H, m, ArH); ^13^C NMR (100 MHz, CDCl_3_, δ_C_): 117.0 (s, C-5), 118.1 (s, C-6), 126.0 (s, C-7), 126.8 (s, C-8), 133.4 (dd, ^2^*J*_CF_ = 6.3 Hz, ^3^*J*_CF_ = 6.3 Hz, C-4*a*), 136.8 (dd, ^1^*J*_CF_ = 278.0 Hz, ^2^*J*_CF_ = 30.0 Hz, C-4), 138.6 (s, C-8a), 140.3 (s, C-9a), 154.5 (s, C-10a), 156.9 (dd, ^1^*J*_CF_ = 290 Hz, ^2^*J*_CF_ = 8.0 Hz, C-3); ^19^F NMR (376 MHz, CDCl_3_, δ_F_): −96.4 (1F, d, ^3^*J*_FF_ = 25.8 Hz, F-3), −151.9 (1F, d, ^3^*J*_FF_ = 25.9 Hz, F-4); MS (EI^+^) *m/z*: 222 ([M]^+^, 10%), 138 (43), 74 (66), 63 (67), 50 (100).

### Reaction of 3,4-difluoro-9,10-dioxa-1,2-diaza-anthracene (**5**) with morpholine

#### 3-Fluoro-4-(morpholin-4-yl)-9,10-dioxa-1,2-diaza-anthracene (**9a**)

A mixture of 3,4-difluoro-9,10-dioxa-1,2-diaza-anthracene (**5**) (0.20 g, 0.90 mmol), morpholine (0.16 mL, 1.80 mmol) and acetonitrile (2 mL) were placed in a 0.5–2 ml microwave vial under an argon atmosphere and subjected to microwave irradiation at 150 °C for 20 min. The mixture was partitioned between dichloromethane (20 mL) and water (20 mL) and the organic layer separated. The aqueous layer was then extracted with dichloromethane (3 × 20 mL) to give a crude yellow material. Column chromatography on silica gel using hexane:ethyl acetate (2:1) as eluent gave 3-fluoro-4-(morpholin-4-yl)-9,10-dioxa-1,2-diaza-anthracene (**9a**) (0.18 g, 71%) as white crystals; mp 207–208 °C; Found: [MH]^+^, 290.09345. C_14_H_12_FN_3_O_3_ requires: [MH]^+^, 290.09355; ^1^H NMR (700 MHz, CDCl_3_, δ_H_): 3.44 (4H, t, ^3^*J*_HH_ = 4.4 Hz, H-2′), 3.84 (4H, t, ^3^*J*_HH_ = 4.4 Hz, H-3′), 6.94 (1H, d, ^3^*J*_HH_ = 7.6 Hz, ArH), 7.01 (1H, tm, ^3^*J*_HH_ = 7.6 Hz, ArH), 7.06 (2H, m, ArH); ^13^C NMR (175 MHz, CDCl_3_, δ_C_): 50.4 (d, ^4^*J*_CF_ = 4.0 Hz, C-2′), 67.3 (s, C-3′), 116.5 (s, C-5), 117.7 (s, C-8), 125.2 (s, C-6), 125.9 (s, C-7), 126.0 (d, ^2^*J*_CF_ = 25.4 Hz, C-4), 134.6 (d, ^3^*J*_CF_ = 8.9 Hz, C-4*a*), 139.4 (s, C-8*a*), 140.9 (s, C-10*a*), 153.7 (s, C-9*a*), 159.0 (d, ^1^*J*_CF_ = 237.7 Hz, C-3); ^19^F NMR (658 MHz, CDCl_3_, δ_F_): −86.4 (s); MS (ES^+^) *m/z*: 290 ( [MH]^+^, 100%).

### Reaction of 3,4-difluoro-9,10-dioxa-1,2-diaza-anthracene (**5**) with allylamine

#### 4-Allylamino-3-fluoro-9,10-dioxa-1,2-diaza-anthracene (**9b**)

Using the procedure described above, 3,4-difluoro-9,10-dioxa-1,2-diaza-anthracene (**5**) (0.15 g, 0.67 mmol), allylamine (0.10 mL, 1.35 mmol) and acetonitrile (2 mL) gave 4-allylamino-3-fluoro-9,10-dioxa-1,2-diaza-anthracene (**9b**) (0.14 g, 80%) as white crystals; mp 175–177 °C; Anal Calcd for C_13_H_10_FN_3_O_2_: C, 60.2; H, 3.9; N, 16.2%. Found: C, 60.3; H, 4.0; N, 16.3. ^1^H NMR (500 MHz, DMSO-*d*_6_, δ_H_): 4.04 (2H, t, ^3^*J*_HH_ = 5.1 Hz, NCH_2_), 5.10 (1H, dd, ^3^*J*_HH_ = 10.3 Hz, ^2^*J*_HH_ = 1.5 Hz, =CH_2_), 5.17 (1H, dd, ^3^*J*_HH_ = 17.2 Hz, ^2^*J*_HH_ = 1.5 Hz, =CH_2_), 5.94 (1H, ddt, ^3^*J*_HH_ = 17.2 Hz, 10.2, 5.1, -CH=), 6.96 (1H, br t, ^3^*J*_HH_ = 5.1 Hz, NH), 7.07 (3H, m, ArH), 7.12 (1H, m, ArH); ^13^C NMR (125 MHz, DMSO-d_6_, δ_C_): 45.7 (d, ^4^*J*_CF_ = 2.5 Hz, NCH_2_), 115.3 (s, =CH_2_), 116.4 (s, C-5), 117.0 (s, C-6), 124.6 (d, ^2^*J*_CF_ = 28.2 Hz, C-4), 125.1 (s, C-7), 125.2 (s, C-8), 127.2 (d, ^3^*J*_CF_ = 9.6 Hz, C-4*a*), 136.1 (s, CH=), 139.4 (s, C-8*a*), 140.5 (s, C-10*a*), 152.3 (s, C-9*a*), 155.2 (d, ^1^*J*_CF_ = 230.4 Hz, C-3); ^19^F NMR (470 MHz, DMSO-*d*_6_, δ_F_) −93.7 (s); MS (ES^+^) *m/z*: 323 ([M+MeCN+Na]^+^, 100%), 260 ([MH]^+^, 68), 219 (69).

*Crystal data for* **9b**: C_13_H_10_FN_3_O_2_, M = 259.24, monoclinic, space group *P*2_1_/*n*, a = 4.9065(1), b = 19.5663(4), c = 11.8180(2) Å, β = 94.25(1)°, U = 1131.44(4) Å^3^, F(000) = 536, Z = 4, *D*_c_ = 1.5220 mg·m^−3^, μ = 0.117 mm^−1^ (Mo Kα, λ = 0.71073 Å), *T* = 120.0(2) K. 14166 reflections were collected on a Bruker SMART 6000 diffractometer (ω-scan, 0.3°/frame) yielding 2875 unique data (R_merg_ = 0.0615). The structure was solved by direct method and refined by full-matrix least squares on F^2^ for all data using Olex2 software. All non-hydrogen atoms were refined with anisotropic displacement parameters, H-atoms were located on the difference map and refined isotropically. Final wR_2_(F^2^) = 0.1275 for all data (212 refined parameters), conventional R(F) = 0.0439 for 1918 reflections with I ≥ 2σ, GOF = 0.985. Crystallographic data for the structure have been deposited with the Cambridge Crystallographic Data Centre as supplementary publication CCDC-764716.

### Reaction of 3,4-difluoro-9,10-dioxa-1,2-diaza-anthracene (**5**) with sodium ethoxide

#### 4-Ethoxy-3-fluoro-9,10-dioxa-1,2-diaza-anthracene (**9c**)

Using the procedure described above, 3,4-difluoro-9,10-dioxa-1,2-diaza-anthracene (**5**) (0.10 g, 0.45 mmol), sodium ethoxide (0.06 g, 0.90 mmol) and ethanol (2 mL) gave 4-ethoxy-3-fluoro-9,10-dioxa-1,2-diaza-anthracene (**9c**) (0.07 g, 66%), as white crystals; mp 131–133 °C; Anal Calcd for C_12_H_9_FN_2_O_3_: C, 58.1; H, 3.7; N, 11.3%. Found: C, 58.0; H, 3.7; N, 11.2. ^1^H NMR (500 MHz, CDCl_3_, δ_H_): 1.49 (3H, t, ^3^*J*_HH_ = 7.0 Hz, CH_3_), 4.52 (2H, qd, ^3^*J*_HH_ = 7.0 Hz, ^5^*J*_HF_ = 1.4 Hz, OCH_2_), 7.00–7.11 (4H, m, ArH); ^13^C NMR (125 MHz, DMSO-d_6_, δ_C_): 15.7 (s, CH_3_), 70.6 (d, ^4^*J*_CF_ = 3.9 Hz, OCH_2_), 116.7 (s, C-5), 117.8 (s, C-6), 125.4 (s, C-7), 126.1 (s, C-8), 133.6 (d, ^2^*J*_CF_ = 27.2 Hz, C-4), 135.1 (d, ^3^*J*_CF_ = 8.2 Hz, C-4*a*), 139.2 (s, C-8*a*), 140.7 (s, C-10*a*), 154.0 (d, ^4^*J*_CF_ = 1.5 Hz, C-9*a*), 158.2 (d, ^1^*J*_CF_ = 239.5 Hz, C-3); ^19^F NMR (470 MHz, CDCl_3_, δ_F_) −92.4 (s); MS (ES^+^) *m/z*: (249 ([MH]^+^), 100%).

## Supporting Information

Supporting Information with ^1^H NMR and ^13^C NMR spectra for 3,4,6-trifluoro-5-phenoxypyridazine (**7**), ^1^H NMR, ^13^C NMR and ^19^F NMR spectra for 3,6-difluoro-4,5-diphenoxypyridazine (**8**), 3,4-difluoro-9,10-dioxa-1,2-diaza-anthracene (**5**), 3-fluoro-4-(morpholin-4-yl)-9,10-dioxa-1,2-diaza-anthracene (**9a**), 4-allylamino-3-fluoro-9,10-dioxa-1,2-diaza-anthracene (**9b**), 4-ethoxy-3-fluoro-9,10-dioxa-1,2-diaza-anthracene (**9c**).

File 1 NMR spectra of all synthesized compounds **7, 8, 5** and **9a–9c**
