# Supplementary material for: 9,10-Dioxa-1,2-diaza-anthracene derivatives from tetrafluoropyridazine
Source: Beilstein J Org Chem. 2010 May 6;6:45. doi: 10.3762/bjoc.6.45 (PMC2887278; doi:10.3762/bjoc.6.45)
Supplement: File 1 — NMR spectra of all synthesized compounds 7, 8, 5 and 9a–9c [file Beilstein_J_Org_Chem-06-45-s001.pdf]

Supporting Information to

9,10-Dioxa-1,2-diaza-anthracene derivatives from tetrafluoropyridazine

Graham Pattison[1], Graham Sandford[*1], Dmitrii S. Yufit[2], Judith A. K. Howard[2], John A. Christopher[3] and David D. Miller[3]

Address: [1]Department of Chemistry, University of Durham, South Road, Durham, DH1 3LE, United Kingdom, [2]Chemical Crystallography Group, Department of Chemistry, University of Durham, South Road, Durham, DH1 3LE, U.K. and [3]GlaxoSmithKline R&D, Medicines Research Centre, Gunnels Wood Road, Stevenage, Hertfordshire, SG1 2NY, United Kingdom

Email: Graham Sandford – Graham.Sandford@durham.ac.uk

* Corresponding author

NMR spectra of all synthesized compounds 7, 8, 5 and 9a–9c

^{1}H NMR and ^{13}C NMR spectra for 3,4,6-trifluoro-5-phenoxypyridazine (**7**);

^{1}H NMR, ^{13}C NMR and ^{19}F NMR spectra for 3,6-difluoro-4,5-diphenoxypyridazine (**8**), 3,4-difluoro-9,10-dioxa-1,2-diaza-anthracene (**5**), 3-fluoro-4-(morpholin-4-yl)-9,10-dioxa-1,2-diaza-anthracene (**9a**), 4-allylamino-3-fluoro-9,10-dioxa-1,2-diaza-anthracene (**9b**), 4-ethoxy-3-fluoro-9,10-dioxa-1,2-diaza-anthracene (**9c**).

3,4,6-Trifluoro-5-phenoxypyridazine (7)

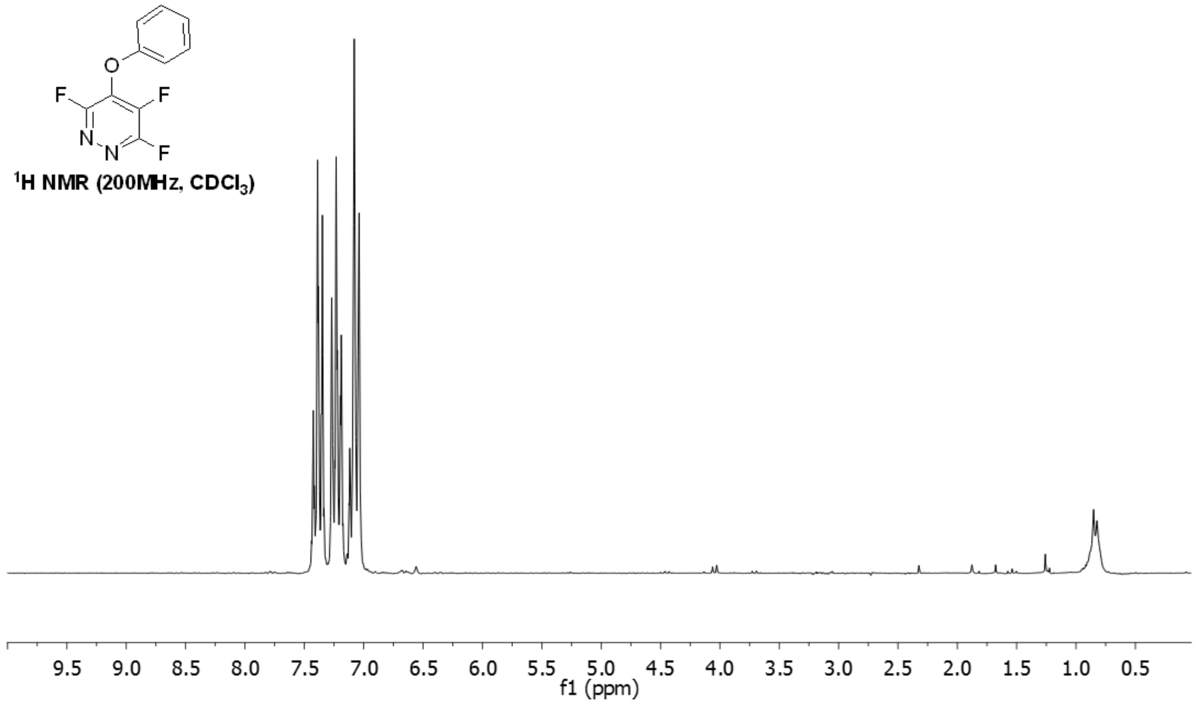

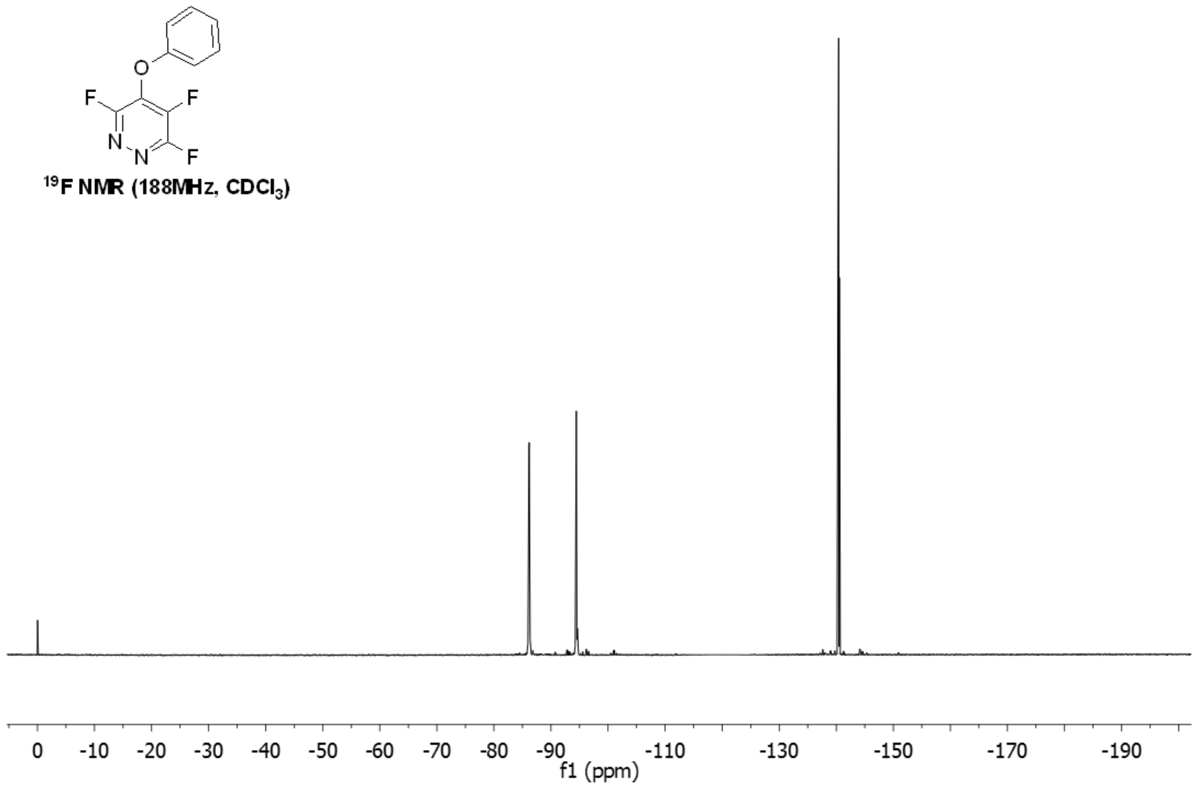

3,6-Difluoro-4,5-diphenoxypyridazine (8)

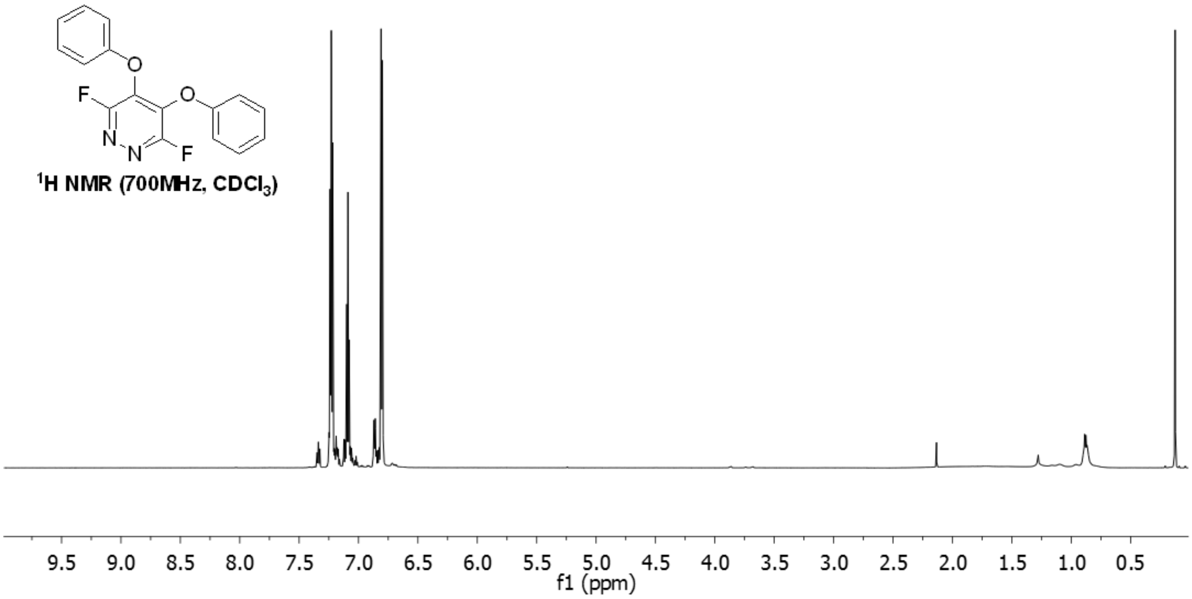

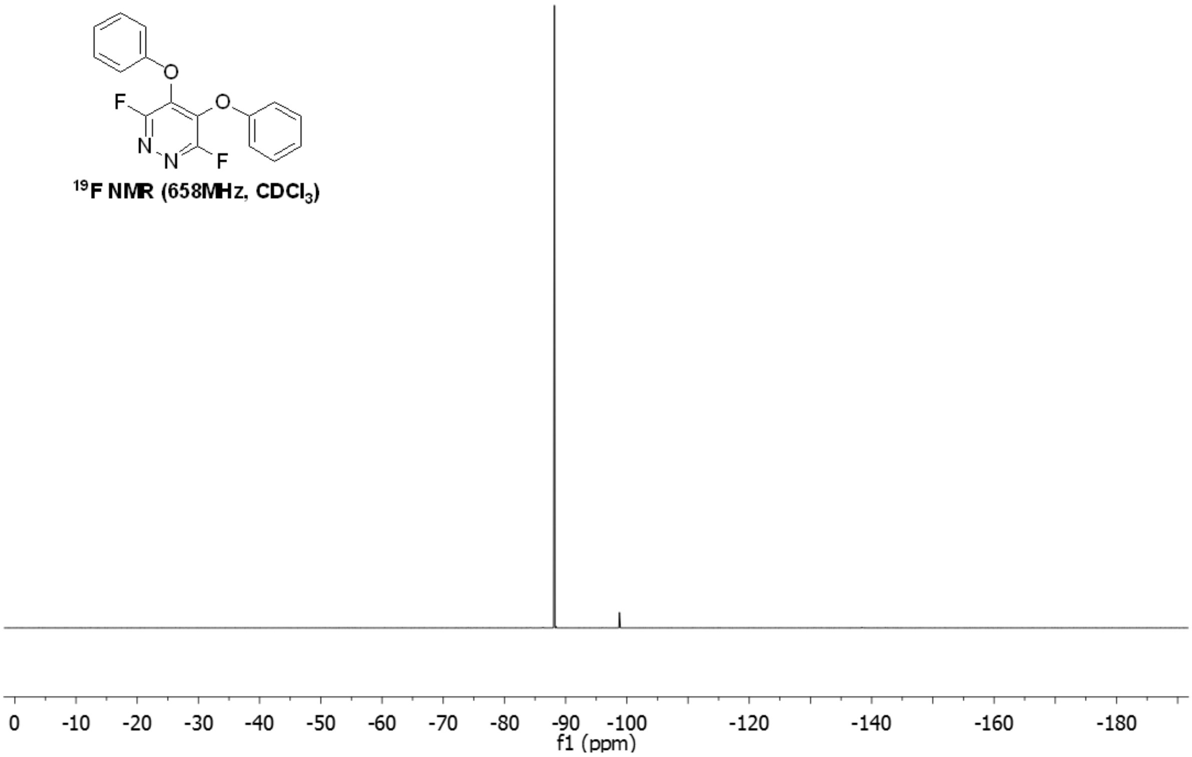

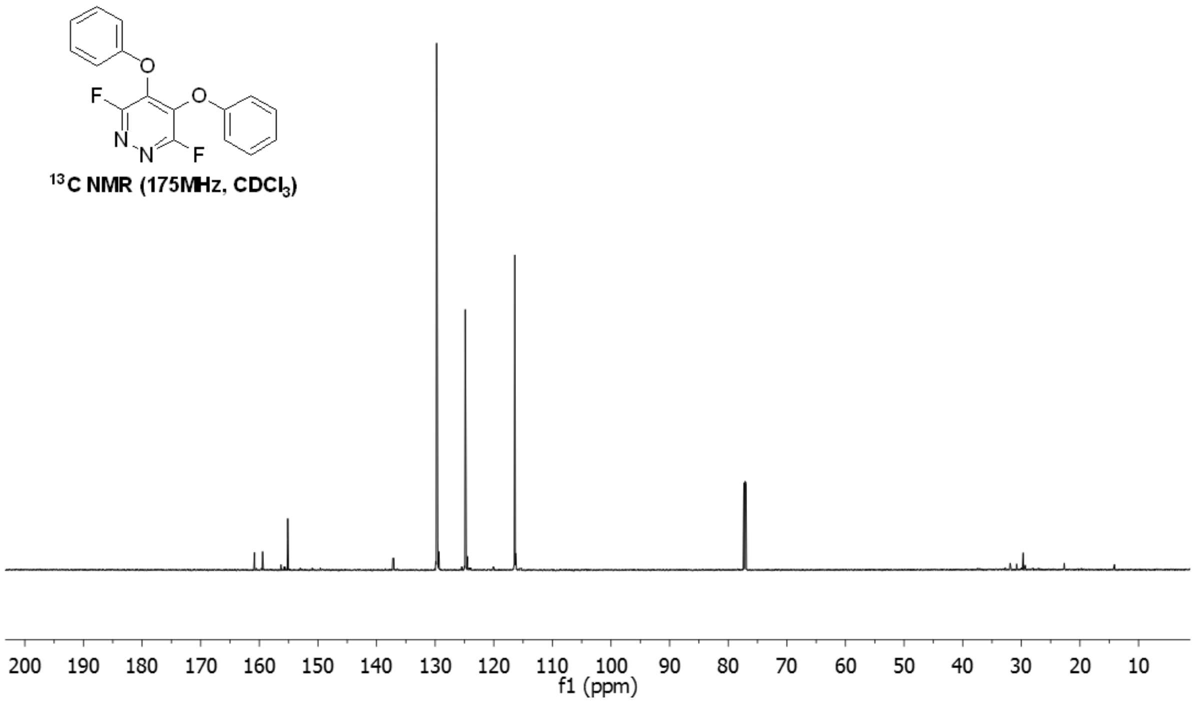
F
N
N
F
O
O
13C NMR (175MHz, CDCl3)
200 190 180 170 160 150 140 130 120 110 100 90 80 70 60 50 40 30 20 10
f1 (ppm)

3,4-Difluoro-9,10-dioxa-1,2-diaza-anthracene (5)

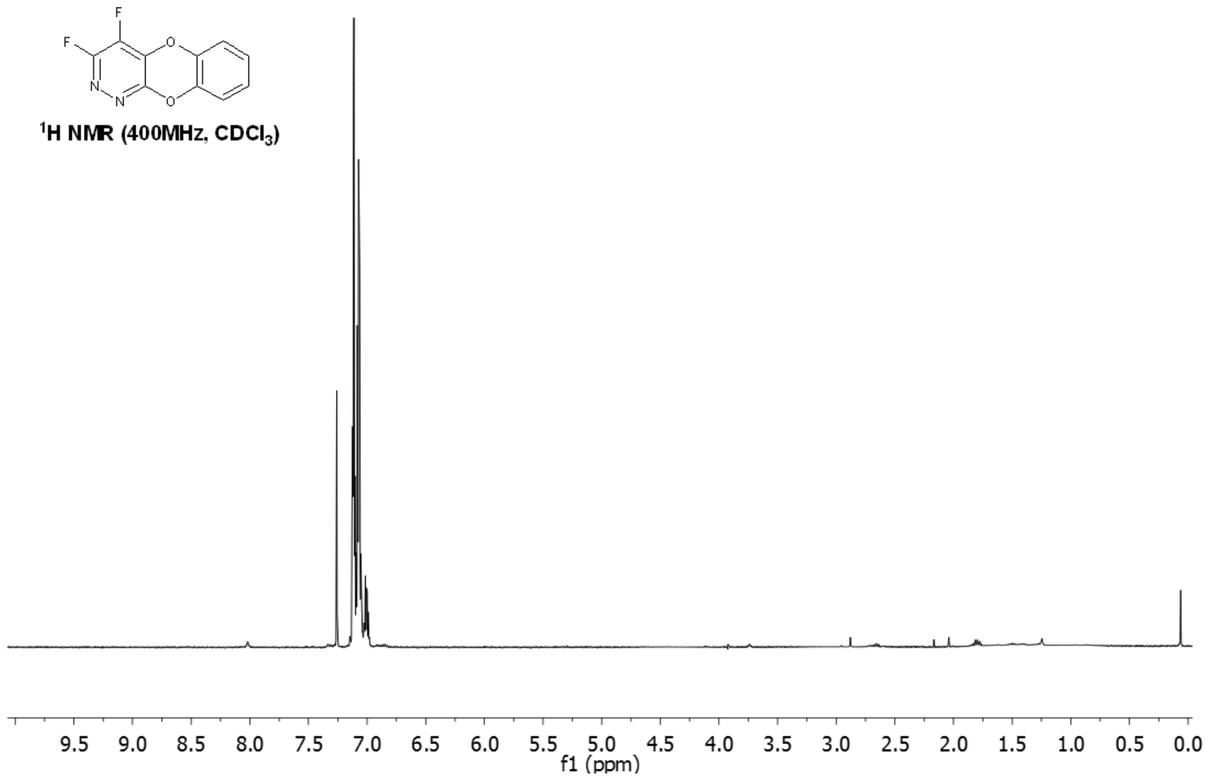

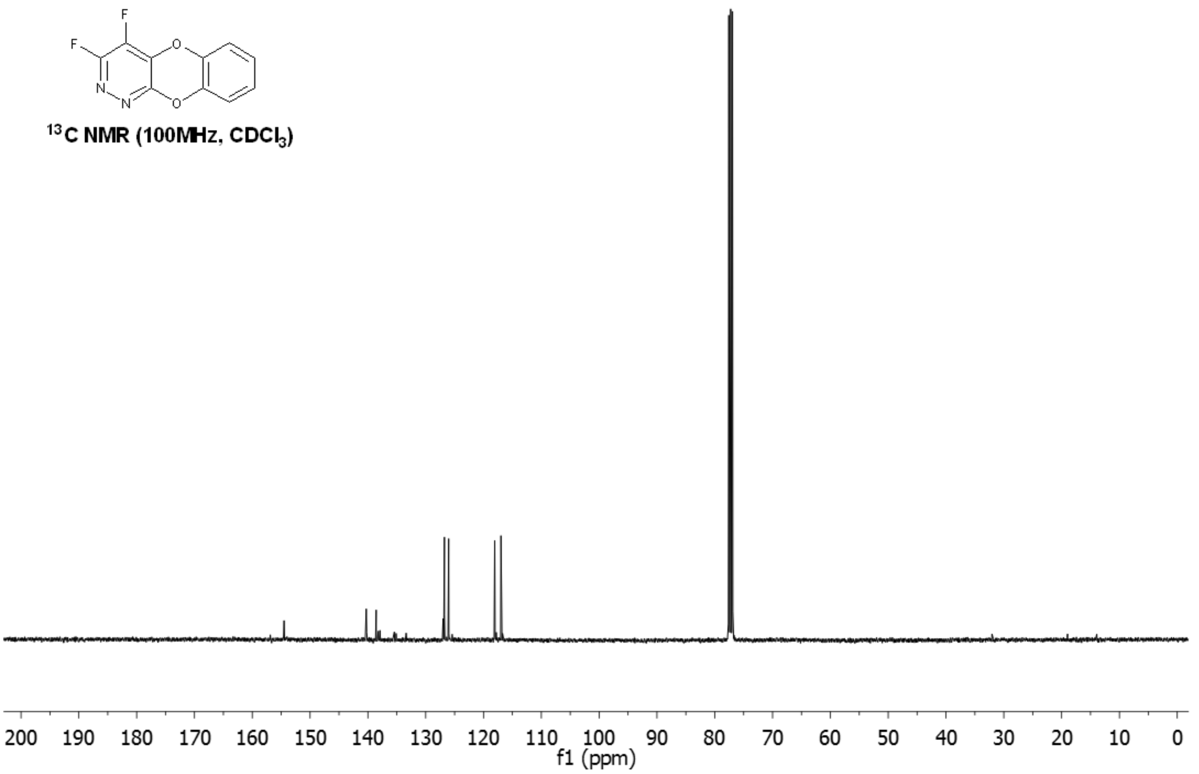

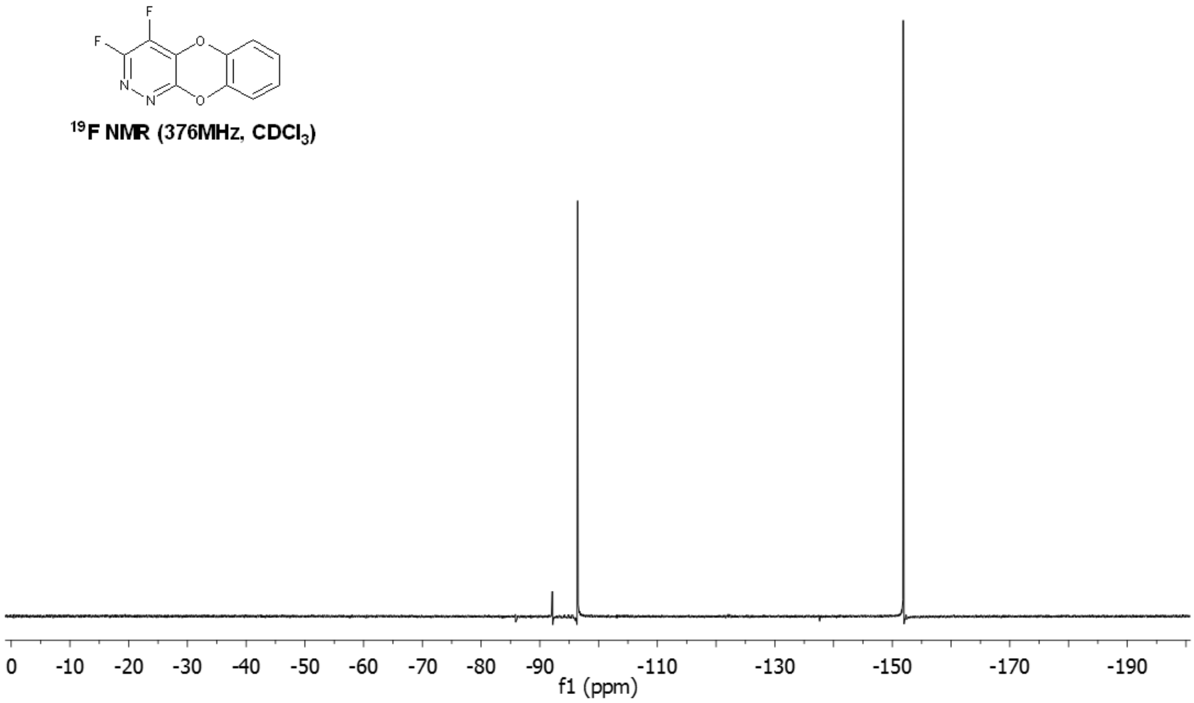
F
F
N
N
O
O
^{19}F NMR (376MHz, $CDCl_3$)
0 -10 -20 -30 -40 -50 -60 -70 -80 -90 -110 -130 -150 -170 -190
f1 (ppm)

3-Fluoro-4-(morpholin-4-yl)-9,10-dioxa-1,2-diaza-anthracene (9a)

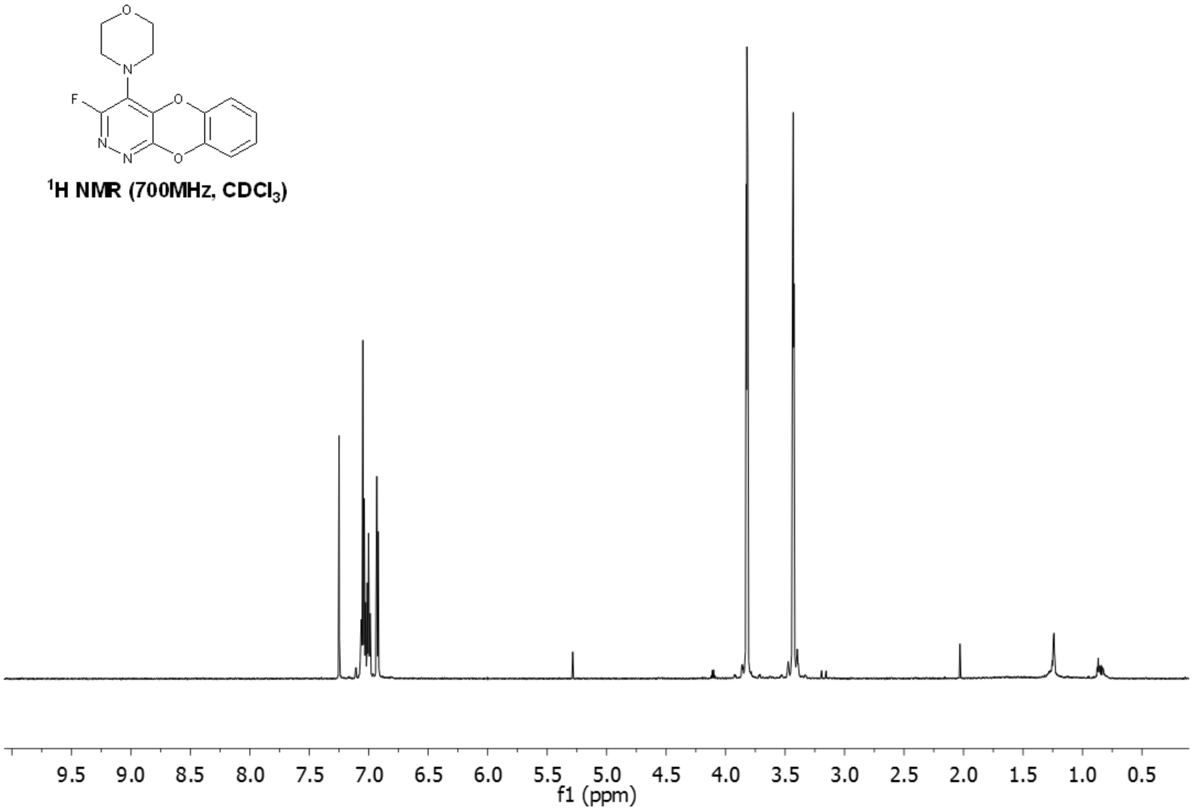

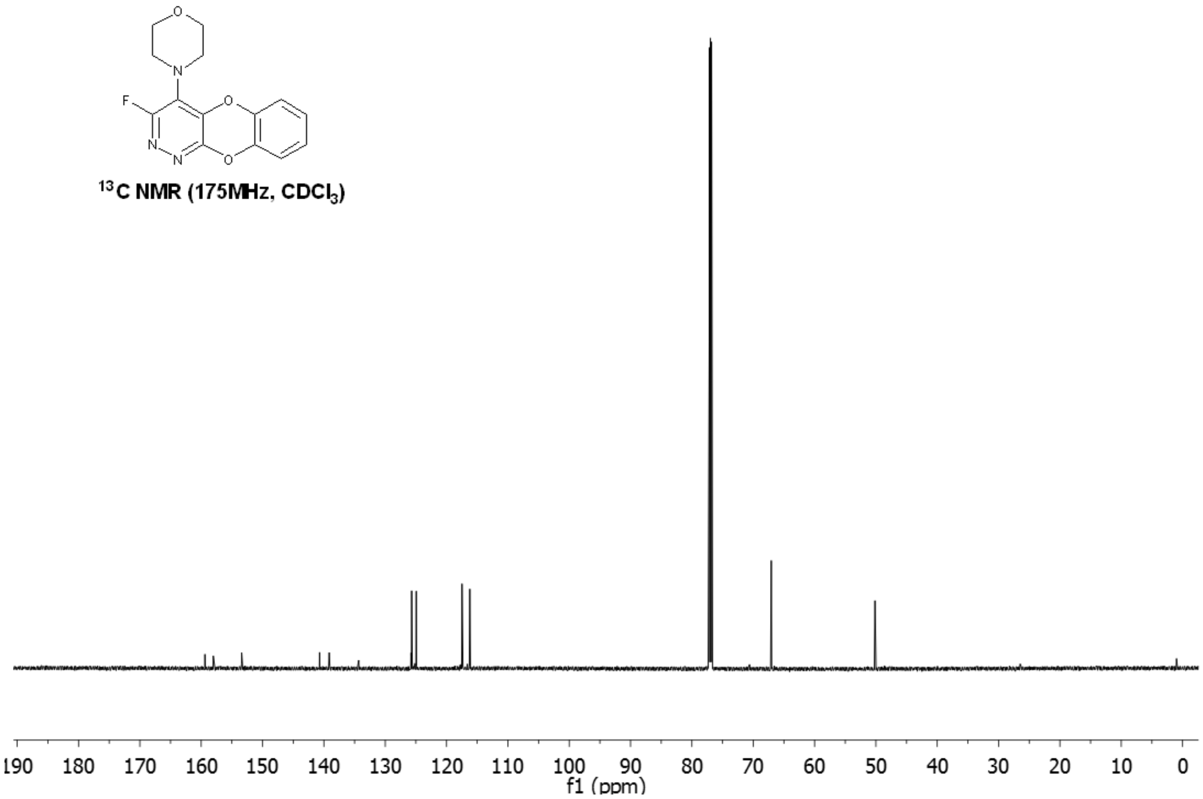

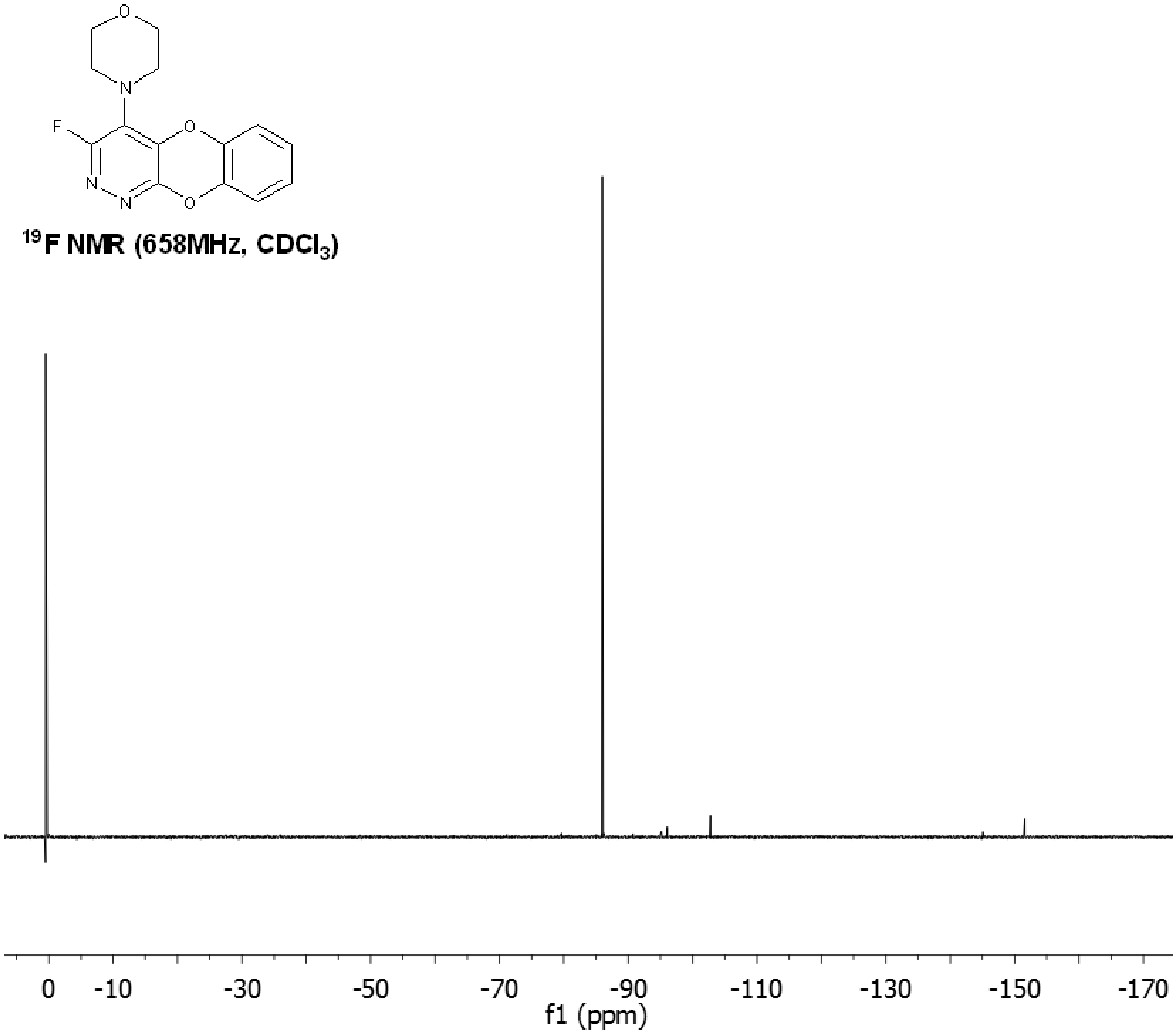

^{19}F NMR (658MHz, $CDCl_3$)
0 -10 -30 -50 -70 -90 -110 -130 -150 -170
f1 (ppm)

4-Allylamino-3-fluoro -9,10-dioxa-1,2-diaza-anthracene (9b)

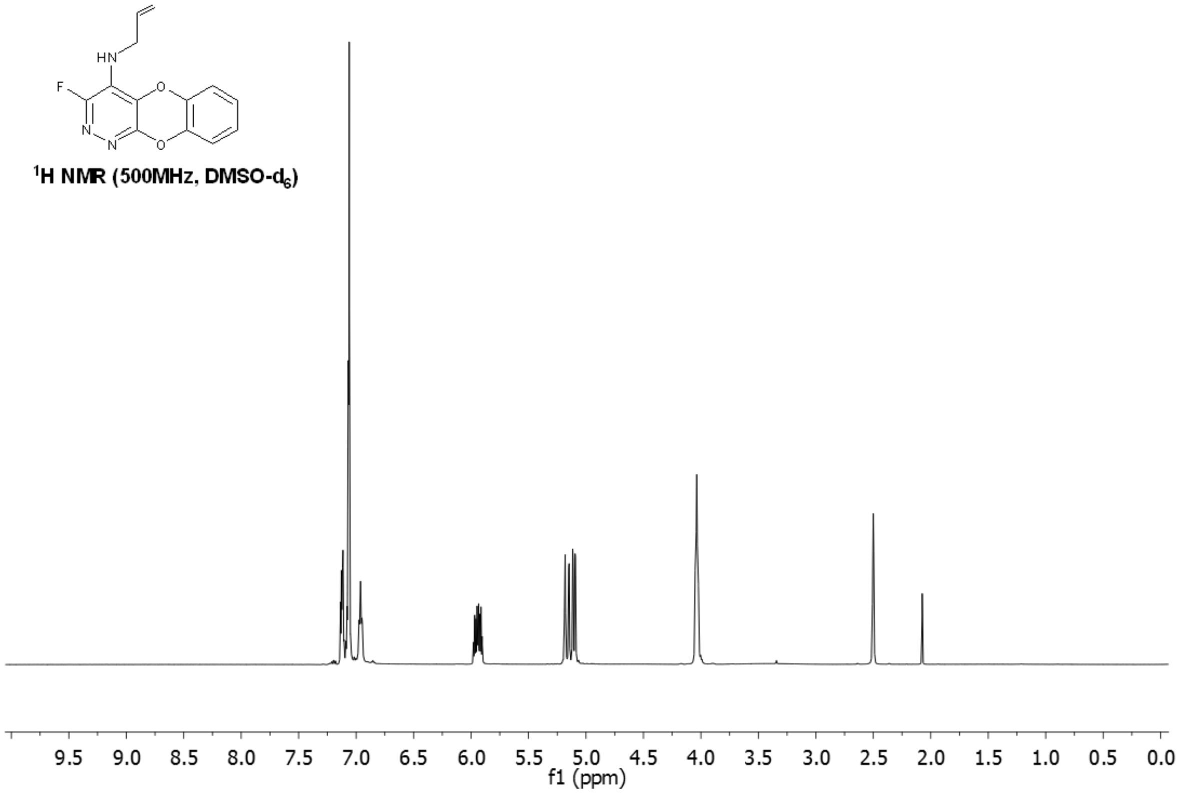

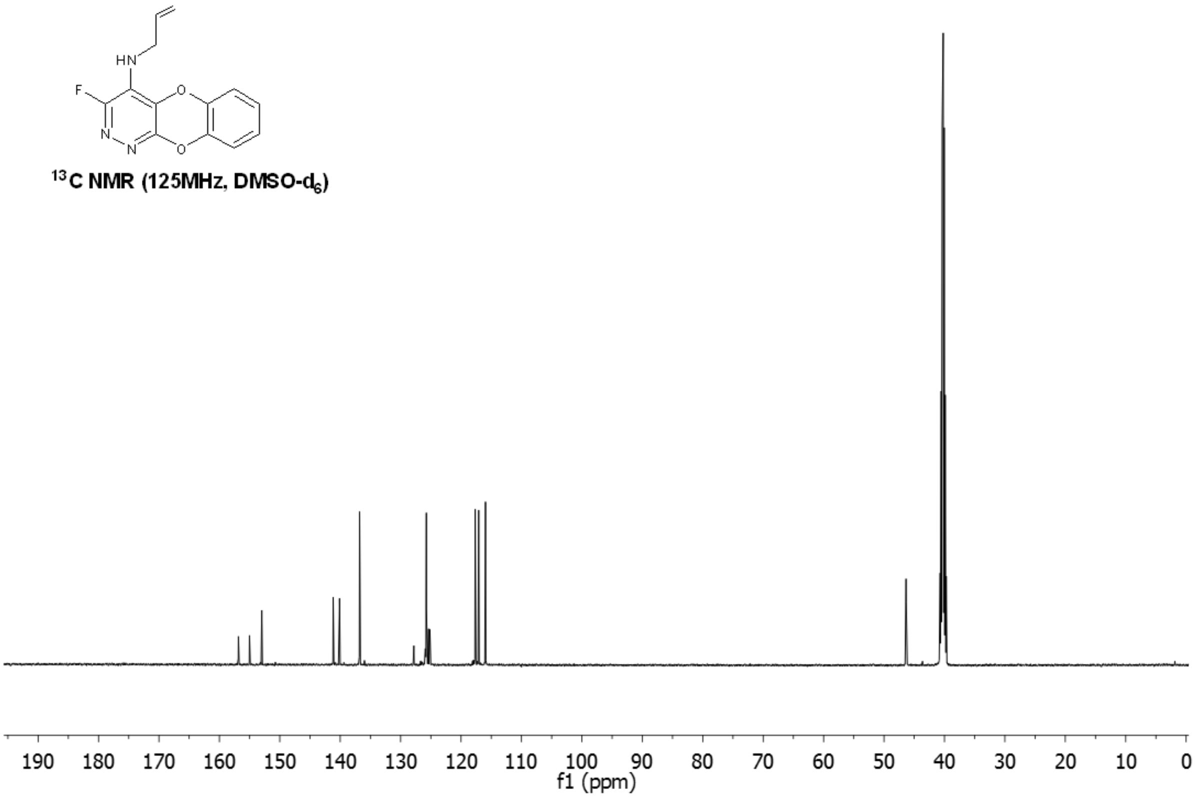

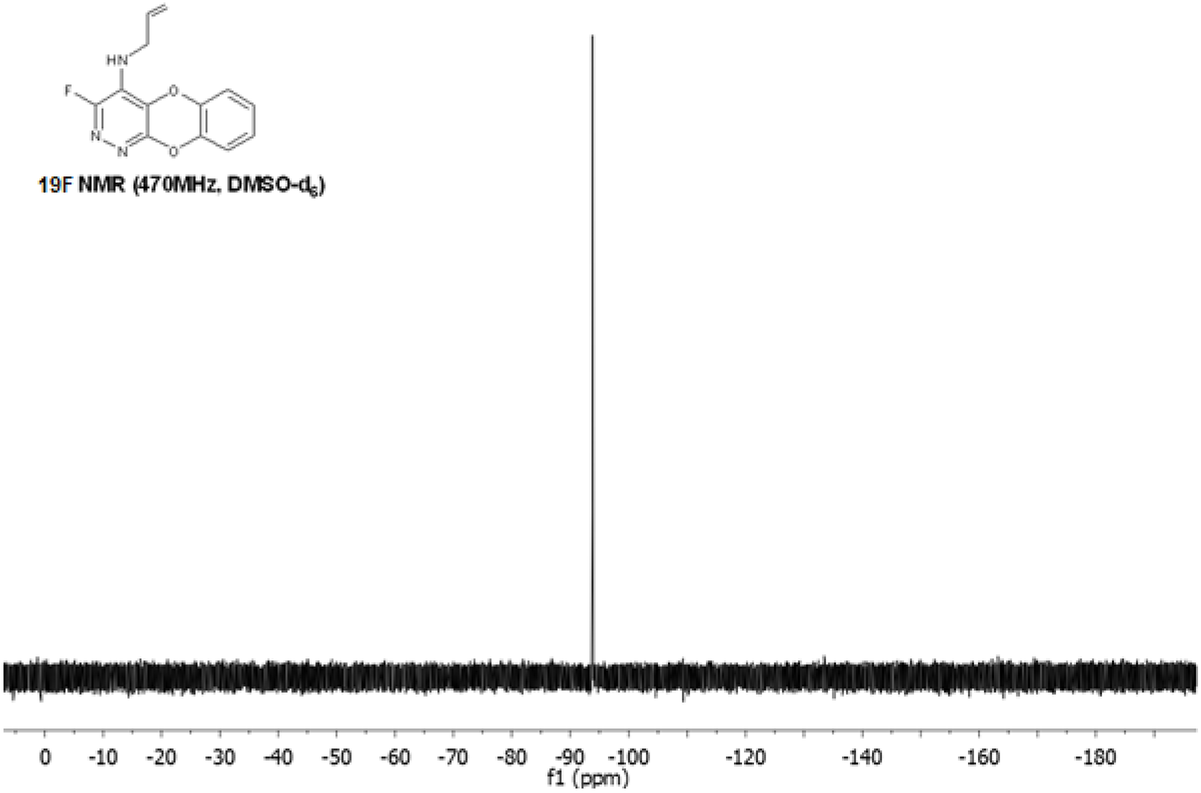
HN
F
N
N
O
O
19F NMR (470MHz, DMSO-d$_6$)
0
-10
-20
-30
-40
-50
-60
-70
-80
-90
-100
-120
-140
-160
-180
f1 (ppm)

4-Ethoxy-3-fluoro-9,10-dioxa-1,2-diaza-anthracene (9c)

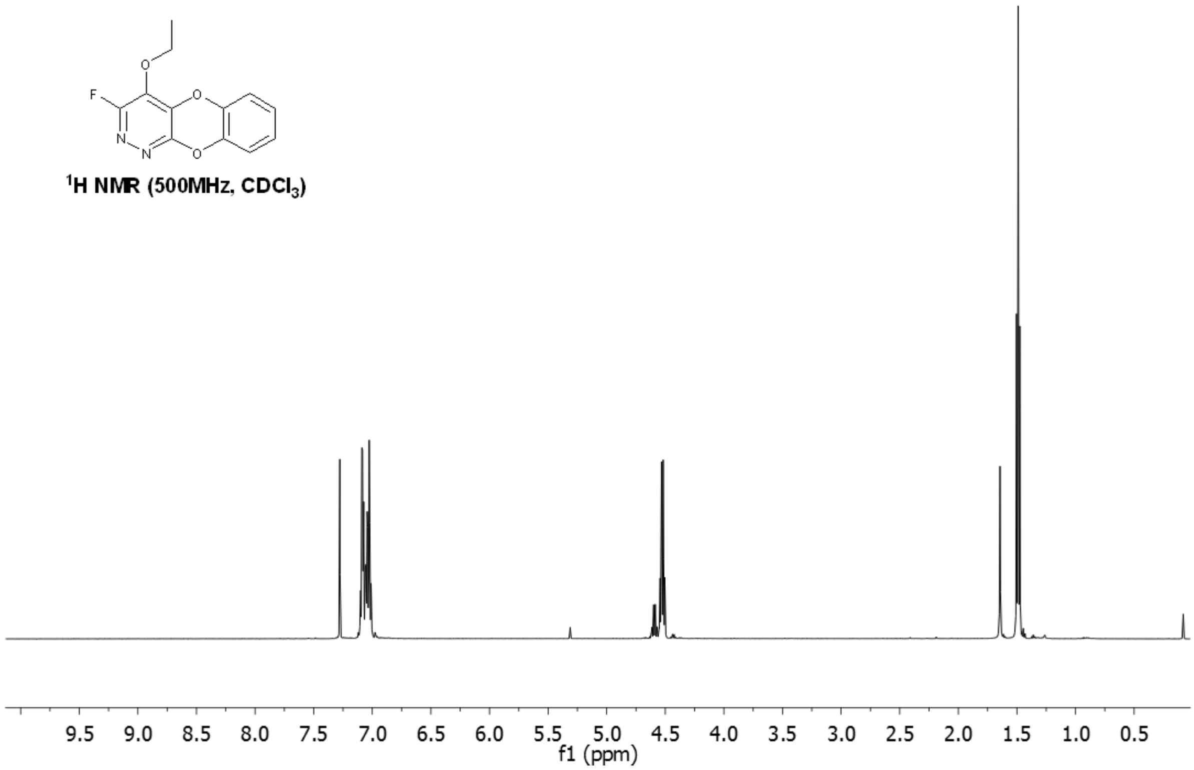

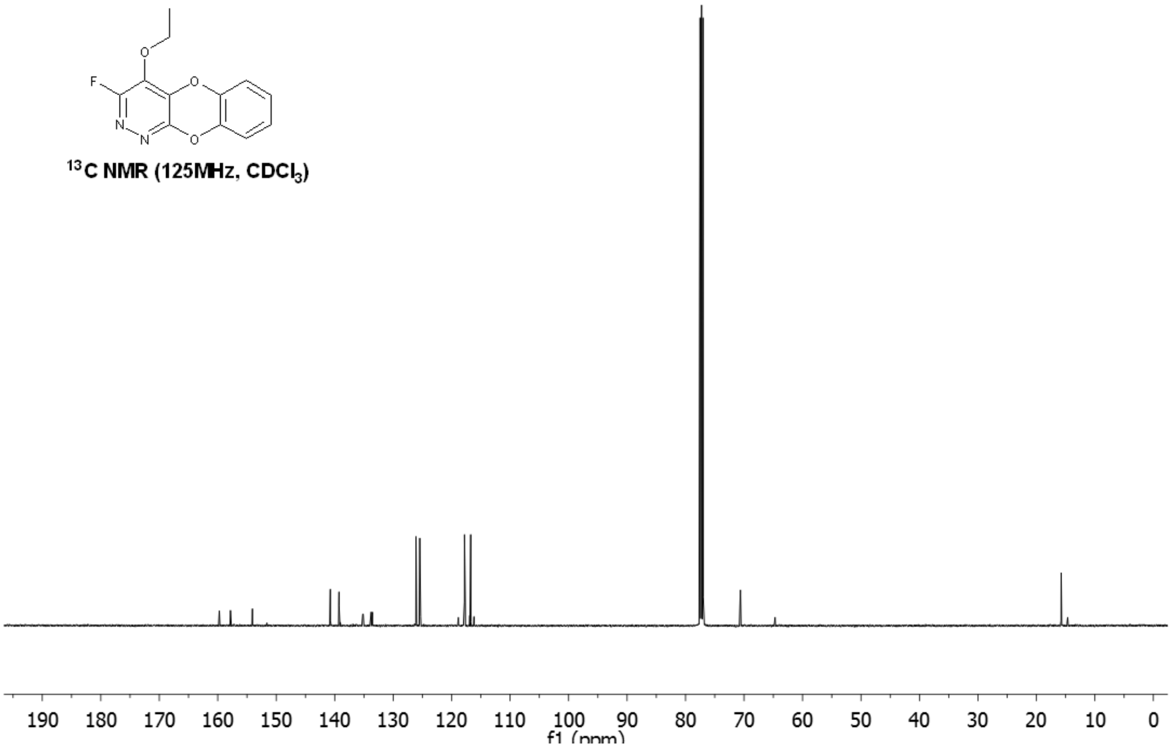

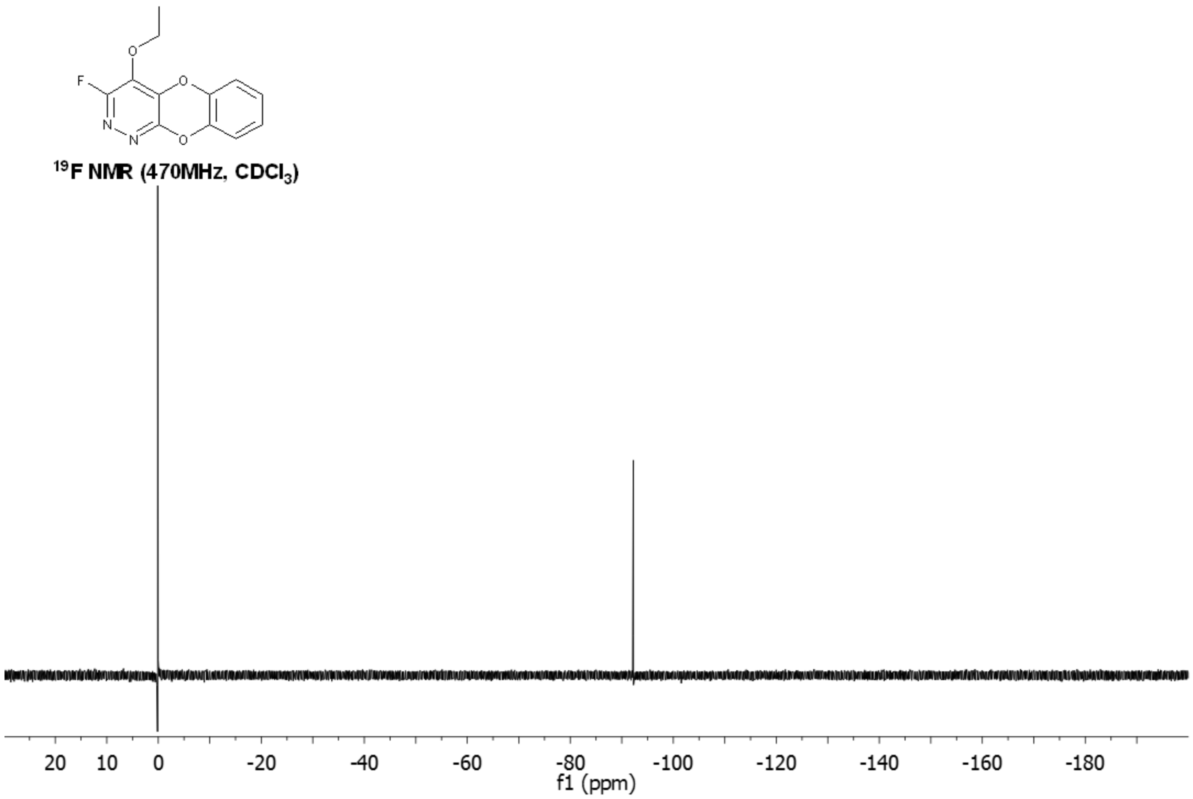
F
O
N
N
O
O
^{19}F NMR (470MHz, CDCl$_3$)
20 10 0 -20 -40 -60 -80 -100 -120 -140 -160 -180
f1 (ppm)
